# Supplementary material for: A pseudovirus system enables deep mutational scanning of the full SARS-CoV-2 spike
Source: Cell. 2023 Mar 16;186(6):1263–1278.e20. doi: 10.1016/j.cell.2023.02.001 (PMC9922669; doi:10.1016/j.cell.2023.02.001)
Supplement: Table S1. Primer sequences used for building deep mutational scanning libraries, related to STAR Methods sections on the production of deep mutational scanning libraries [file mmc1.pdf]

Cell, Volume *186*

Supplemental information

A pseudovirus system enables deep mutational scanning of the full SARS-CoV-2 spike

Bernadeta Dadonaite, Katharine H.D. Crawford, Caelan E. Radford, Ariana G. Farrell, Timothy C. Yu, William W. Hannon, Panpan Zhou, Raiees Andrabi, Dennis R. Burton, Lihong Liu, David D. Ho, Helen Y. Chu, Richard A. Neher, and Jesse D. Bloom

Supplementary table 1

**Name**	**Sequence 5′-3′**	**Function**
Mutagenesis primers		
BA.1 oPools	https://github.com/dms-vep/SARS-CoV-2_Omicron_BA.1_spike_DMS_mAbs/tree/main/library_design	Primers to add mutations to BA.1 spike
Delta oPools	https://github.com/dms-vep/SARS-CoV-2_Delta_spike_DMS_REGN10933/tree/main/library_design/results	Primers to add mutations to Delta spike
Spike amplification and joining PCR		
VEP_amp_for	CAGCCGAGCCACATCGCTC	For amplifying spike with flanking sequences from lentivirus backbone
3’rev_lib_LinJoin_KHDC	CGGAAGAGCGTCGTGTAGGGAAAG	
Spike barcoding primers		
ForInd_AddBC_2	gcggaactccactaggaacatttctctctcgaaTCTAGANNNNNNNNNNNNNNNNAGATCGGAAGAGCGTCGTGTAGGGAAAGAG	For adding barcodes to spike gene
5'for_lib_bcing	gcacgcgCAGCCGAGCCACATCGCTCA	
Neutralization standard barcoding primers		
5'for_lib_bcing	gcacgcgCAGCCGAGCCACATCGCTCA	For barcoding neutralization satandard
Barcoding primer pool 1:<br>VSVG_BC1<br>VSVG_BC2<br>VSVG_BC3<br>VSVG_BC4	gcggaactccactaggaacatttctctctcgaaTCTAGAtactttactactgcacAGATCGGAAGAGCGTCGTGTAGGGAAAGAG<br>gcggaactccactaggaacatttctctctcgaaTCTAGAggaccattgcgacgtaAGATCGGAAGAGCGTCGTGTAGGGAAAGAG<br>gcggaactccactaggaacatttctctctcgaaTCTAGAcctagccactagatggAGATCGGAAGAGCGTCGTGTAGGGAAAGAG<br>gcggaactccactaggaacatttctctctcgaaTCTAGAatggagggagtctactAGATCGGAAGAGCGTCGTGTAGGGAAAGAG	
Barcoding primer pool 2<br>VSVG_BC5<br>VSVG_BC6<br>VSVG_BC7<br>VSVG_BC8	gcggaactccactaggaacatttctctctcgaaTCTAGAtagtgtaaacgccacgAGATCGGAAGAGCGTCGTGTAGGGAAAGAG<br>gcggaactccactaggaacatttctctctcgaaTCTAGAccaacgcgtgaatcgcAGATCGGAAGAGCGTCGTGTAGGGAAAGAG<br>gcggaactccactaggaacatttctctctcgaaTCTAGAatcgtatccatgggtaAGATCGGAAGAGCGTCGTGTAGGGAAAGAG<br>gcggaactccactaggaacatttctctctcgaaTCTAGAggtcacgtgtctatatAGATCGGAAGAGCGTCGTGTAGGGAAAGAG	
Spike gene amplification for PacBio long-read sequencing		
PacBio_5pri_C_tag1	ctagccattcagagGCAGCCGAGCCACcTCGCTC	Used for amplifying spike and barcode for
PacBio_3pri_G_tag1	CGCTCAACCAGTACGAGCCGTAAGTTATGTAACGCGGAACTCCACgAGGAAC	

PacBio_5pri_G_tag2	ctagccattcagagGCAGCCGAGCCACgTCGCTC	PacBio sequencing
PacBio_3pri_C_tag2	CGCTCAACCAGTACGAGCCGTAAGTTATGTAACGCGGAACTCCACcAGGAAC	
PacBio_5pri_RND2	CTAGCCATTCAGAGGCAGCCGAG	
PacBio_3pri_RND2	CGCTCAACCAGTACGAGCCGTAAGTTATGTAAC	
Illumina barcode sequencing 1st round PCR primers		
IlluminaRnd1_For	CTCTTTCCCTACACGACGCTCTTCCGATCT	Round 1 primers for barcode sequencing
IlluminaRnd1_Rev3	CTGGAGTTCAGACGTGTGCTCTTCCGATCTgtccctattggcgttactatgggaacatacgtc	
Illumina barcode sequencing library round 2 PCR primers		
Rnd2ForUniversal	AATGATACGGCGACCACCGAGATCTACACTCTTTCCCTACACGACGCTCTTCCGATCT	Universal illumina primer and indexing primer, where xxxxxxx indicates i7 index sequence
Indexing primer	CAAGCAGAAGACGGCATACGAGATxxxxxxxxGTGACTGGAGTTCAGACGTGTGCTCTTCCGATCT	
